# Genomes and Virulence Factors of Novel Bacterial Pathogens Causing Bleaching Disease in the Marine Red Alga *Delisea pulchra*

**DOI:** 10.1371/journal.pone.0027387

**Published:** 2011-12-05

**Authors:** Neil Fernandes, Rebecca J. Case, Sharon R. Longford, Mohammad R. Seyedsayamdost, Peter D. Steinberg, Staffan Kjelleberg, Torsten Thomas

**Affiliations:** 1 The Centre for Marine Bio-Innovation (CMB), University of New South Wales, Sydney, Australia; 2 School of Biotechnology and Biomolecular Sciences, University of New South Wales, Sydney, Australia; 3 Department of Microbiology and Molecular Genetics, Harvard Medical School, Boston, Massachusetts, United States of America; 4 School of Biological, Earth and Environmental Sciences, University of New South Wales, Sydney, Australia; 5 Department of Biological Chemistry and Molecular Pharmacology, Harvard Medical School, Boston, Massachusetts, United States of America; 6 Singapore Centre on Environmental Life Sciences Engineering, Nanyang Technological University, Singapore, Singapore; Rutgers University, United States of America

## Abstract

*Nautella* sp. R11, a member of the marine Roseobacter clade, causes a bleaching disease in the temperate-marine red macroalga, *Delisea pulchra*. To begin to elucidate the molecular mechanisms underpinning the ability of *Nautella* sp. R11 to colonize, invade and induce bleaching of *D. pulchra*, we sequenced and analyzed its genome. The genome encodes several factors such as adhesion mechanisms, systems for the transport of algal metabolites, enzymes that confer resistance to oxidative stress, cytolysins, and global regulatory mechanisms that may allow for the switch of *Nautella* sp. R11 to a pathogenic lifestyle. Many virulence effectors common in phytopathogenic bacteria are also found in the R11 genome, such as the plant hormone indole acetic acid, cellulose fibrils, succinoglycan and nodulation protein L. Comparative genomics with non-pathogenic Roseobacter strains and a newly identified pathogen, *Phaeobacter* sp. LSS9, revealed a patchy distribution of putative virulence factors in all genomes, but also led to the identification of a quorum sensing (QS) dependent transcriptional regulator that was unique to pathogenic Roseobacter strains. This observation supports the model that a combination of virulence factors and QS-dependent regulatory mechanisms enables indigenous members of the host alga's epiphytic microbial community to switch to a pathogenic lifestyle, especially under environmental conditions when innate host defence mechanisms are compromised.

## Introduction

Disease is increasingly viewed as a major factor in marine ecology and its impact is expected to increase with environmental change such as global warming [1], [2]. Several vital habitat-forming organisms, including sea grasses, corals and macroalgae, have experienced devastating mass mortality events caused by disease [3], [4]. Over the last decade, the temperate-marine red macroalga *Delisea pulchra* has also been observed to undergo a bleaching disease in summer [5]. Bleaching in *D. pulchra* begins with the loss of its photosynthetic pigments at restricted sites and is followed by tissue necrosis and death [5], [6], [7]. The habitat of *D. pulchra* off the Sydney coast is under severe environmental stress due to elevated sea surface temperatures brought about by the increased southward migration of the warm East Australian Current (EAC), suggested to be caused by global climate change [8], [9].

*D. pulchra* can suppress surface colonization by marine micro- and macro-organisms through the production of brominated furanones [10], [11]. These secondary metabolites are potent inhibitors of the N-acyl homoserine lactone (AHL) based quorum sensing (QS) used by some bacteria to coordinate transcription within a population, where the phenotype is beneficial to the population. This includes, for example, the expression of colonization, virulence and other functional traits responsible for population or community behaviour in bacteria [6], [12]. We have recently shown that the bacterial strain R11, which was isolated from *D. pulchra*, is able to produce *in vitro* bleaching symptoms that are identical to those observed in the field [7]. Strain R11 can colonize the surface and form substantial biofilms on chemically undefended (i.e. furanone-depleted) *D. pulchra* thalli. Furthermore, at elevated temperature (from 19 to 24°C) bacterial cells penetrate through the epidermal layer and invade algal cells without visible cell wall destruction. This process coincides with localized bleaching of the thallus. The mechanisms of invasion and bleaching are not understood.

Strain R11 was initially assigned to the genus *Ruegeria* [7]. However the recent establishment of the genus *Nautella* requires a reassignment of strain R11 to this genus as it possesses 100% 16S rRNA gene identity to the type strain *Nautella italica* LMG 24365T (Genbank accession number: AM904562) [13]. *N. italica* LMG 24365T and other related isolates were cultured from a marine electroactive biofilm [13]. Strain R11 also shares 100% 16S rRNA gene identity with the marine bacterial isolate *Rhodobacteraceae* No. 63 (Genbank accession number: AB180391.1), which exerts an algaecide effect *in vitro* against several species of red tide microalgae (*Raphidophyceae*) [14]. Ribosomal RNA gene sequences closely related to *N. italica* have recently been detected in the bacterial communities associated with body wall lesions of the sea urchin *Tripneustes gratilla* and heat-stressed juveniles of the coral *Acropora tenuis* [15], [16] as well as coral white plague and black band disease [17], [18]. These observations indicate that strains closely related to *Nautella* sp. R11 could be involved in a wider range of marine diseases than previously anticipated.

From amongst fully sequenced genomes, the closest phylogenetic neighbour of strain R11 is *Phaeobacter gallaeciensis* (<97% 16S rRNA gene sequence similarity) within the abundant marine Roseobacter clade [19], [20]. Roseobacters, a marine group of alpha proteobacteria, are widely studied in marine systems due to the important role they play in nutrient cycling [21]. However, there have been several reports implicating them as pathogens of marine eukaryotes and particular Roseobacter strains have been linked to disease. These include *Ruegeria atlantica*, which produces a compound that lyses the toxin-producing dinoflagellate *Alexandrium catenella*, [21], [22], *Roseovarius crassostreae*, which is the etiological agent of *Roseovarius* Oyster Disease [23] and *Phaeobacter gallaeciensis*, which produces a potent algacide against the microalga *Emiliania huxleyi* [24]. *Roseovarius* species have also been detected in the microbial community associated with lesions in corals, affected by white plague-like and black band diseases [25], [26]. Certain Roseobacter strains are also capable of infecting several red algal *Prionitis* species leading to gall formation in an infection process that appears to be similar to tumorigenesis induced by *Agrobacterium tumefaciens* [27].

To enhance our understanding of the disease mechanisms and ecology of strain R11, we present here the R11 whole genome analysis as well as a comparison of this genome with those of a range of other Roseobacter strains. The R11 genome reveals features indicative of metabolic versatility, strategies for persistence in the environment, as well as complex bacteria-host interactions and numerous genes encoding virulence factors. Moreover, we provide information on the putative key virulence mechanisms by *in vitro* infection assays, comparative genomics across 18 Roseobacter-affiliated bacteria and *de novo* sequencing of a novel *D. pulchra* pathogen, *Phaeobacter* sp. LSS9.

## Methods

### Genome sequencing, assembly, annotation and analysis

Genomic DNA from strain R11 [7] was isolated using a genomic DNA isolation kit (Qiagen Inc) and was sequenced via a modification of the Sanger/pyrosequencing hybrid strategy developed by Goldberg *et al.* [28]. Briefly, 6484 fosmid inserts (38,000 bp +/− 1339) and 25,996 plasmid inserts (3750 bp +/− 250) were end-sequenced with ABI BigDye on an ABI 3730XL sequencer and 221,634 non-paired, pyrosequencing reads were generated on a Roche GS20 sequencer. The paired-end Sanger data was assembled with Phrap using default parameters to build a genome scaffold and pyrosequencing reads were added to this assembly using Crossmatch in Consed [29]. The assembly was manually checked and resulted in two circular contigs corresponding to the chromosome and one plasmid of strain R11. The genome coverage of the assembly was ∼19× and the error rate was estimated by Consed to be less than one error per 500 kbp.

*Phaeobacter* sp. LSS9 was isolated from *D. pulchra* [30] and its genomic DNA was isolated using a genomic DNA isolation kit (Qiagen Inc). A draft genome of *Phaeobacter* sp. LSS9 was generated by pyrosequencing on a FLX Titanium DNA sequencer (Roche, Penzberg, Germany). The 349,353 non-paired reads were assembled with the Newbler assembler (Roche) using a minimum overlap of 20 bp and 80% overlap identity. The resulting assembly was manually checked in Consed. A total of 51 contigs were generated (with 39 greater than 1000 bp in length) resulting in an expected genome size of 4,102,889 bp, an average coverage of 33.6, an N50 value of 248,054 bp and 99.86% of all bases with error rates of one in 10000 or better.

Open reading frames (ORFs) for both genomes were determined with GLIMMER [31] and each ORF was searched against the COG, KEGG, TIGRFam and curated SwissProt databases using an in-house annotation pipeline [32]. For comparative analysis the genome was loaded into IMG-ER [33] and the annotation was checked manually using the tools available in the system. IMG's gene object identifiers are given in brackets in the Results and Discussion section. The two genomes are publicly available through the IMG-ER website (http://img.jgi.doe.gov/cgi-bin/w/main.cgi) under project ID Gi01983 and Gi05401 for strain R11 and LSS9, respectively.

Identification of unique proteins was performed by pairwise blastp and tblastn comparison of the predicted peptides and intergenic regions in each genome. Uniqueness was defined by cut-offs for an E-value of less than 10^−5^ and for percentage identity of greater than 30% in line with previously established definition for comparative analysis of Roseobacter genomes [34]. The selection of strains included in the analysis (Table 1) was based on availability of cultures and genome data, which were downloaded from IGM-ER [33].

**Table 1:** Roseobacter-affiliated strains screened for the ability to cause bleaching and used for comparative genomics (* estimated genome size; ^B^ indicates strains with ability to cause bleach).

Organism	Isolation Source	NCBI Taxon	Genome size (bp)	Genes	Laboratory of Isolation
*Nautella* sp. R11^B^	Surface of red macro alga *Delisea pulchra*, Sydney, AUS	439497	3819746	3569	CMB, UNSW Sydney, Australia.
*Phaeobacter* sp. LSS9^B^	Surface of red macro alga *Delisea pulchra*, Sydney, AUS	681157	4117408*	3170	CMB UNSW Sydney, Australia.
*Dinoroseobacter shibae* DFL 12	Cultures of marine dinoflagellates, Tokyo Bay, JAP	398580	4417868	4271	I. Wagner-Dobler, GBF, Braunschweig, Germany.
*Maricaulis maris* MCS10	Filtered seawater, Washington State, USA	394221	3368780	3138	J. Smit, University of British Columbia, Canada.
*Oceanicaulis alexandrii* HTCC2633	Sargasso Sea, Atlantic Ocean, BATS	314254	3168201	3081	S. Giovannoni, Oregon State University, USA.
*Oceanicola batsensis* HTCC2597	Sargasso Sea, Atlantic Ocean, USA	252305	4437668	4261	S. Giovannoni, Oregon State University, USA.
*Oceanicola granulosus* HTCC2516	Sargasso Sea, Atlantic Ocean, USA	314256	4039111	3855	S. Giovannoni, Oregon State University, USA.
*Phaeobacter gallaeciensis* 2.10	Surface of green alga *Ulva lactuca*, Sydney	383629	4157399	4017	CMB, UNSW Sydney, Australia.
*Phaeobacter gallaeciensis* BS107	Seawater from larval cultures of scallop *Pecten maximus*	391619	4232367	4136	T. Brinkhoff, University Oldenburg, Germany.
*Rhodobacter sphaeroides* 2.4.1	Delft, Holland, and California from enrichment cultures	272943	4603060	4383	S. Kaplan, University of Texas, USA.
*Rhodobacter sphaeroides* ATCC 17025	Delft, Holland, and California from enrichment cultures	349102	4557127	4475	S. Kaplan, University of Texas, USA.
*Rhodobacter sphaeroides* ATCC 17029	Delft, Holland, and California from enrichment cultures	349101	4489380	4268	S. Kaplan, University of Texas, USA.
*Maritimibacter alkaliphilus* HTCC2654	Sargasso Sea, Atlantic Ocean, USA	314271	4529231	4764	S. Giovannoni, Oregon State University, USA.
*Roseovarius nubinhibens* ISM	Surface waters of the Caribbean sea	89187	3668667	3605	M. Moran, University of Georgia, USA.
*Roseovarius* sp. HTCC2601	Sargasso Sea, Atlantic Ocean, USA	314265	5425920	5522	S. Giovannoni, Oregon State University, USA.
*Ruegeria pomeroyi* DSS-3	Coastal Georgia seawater, USA	246200	4601053	4355	M. Moran, University of Georgia, USA.
*Ruegeria* sp. TM1040	Phycosphere of the dinoflagellate *Pfiesteria piscicida*	292414	4153699	3964	R. Belas, University of Maryland, USA.
*Sulfitobacter* sp. EE-36	Salt marsh on the coast of Georgia, USA	25298	3547243	3542	M. Moran, University of Georgia, USA.

### *In vitro* infection assays

The infection assay of laboratory-cultured algae was performed as described by [7] with minor modifications. Briefly, *D. pulchra* spores were grown in bromide-deficient artificial seawater (Br-ASW) for six to 10 weeks to generate chemically undefended (furanone-deficient) thalli. Epiphytic bacteria were removed by overnight treatment with the antibiotics penicillin G (10 µg/ml), streptomycin (10 µg/ml) and kanamycin (20 µg/ml) prior to using the thalli in the assay. The thalli were then rinsed extensively with Br-ASW to remove antibiotics. The Roseobacter strains (Table 1) were grown in Marine Broth 2216 (Becton, Dickinson and Company, Franklin Lakes, NJ USA) at 25°C and 200 rpm for 16 h. Cells were then harvested by centrifugation at 4°C and washed three times with Br-ASW. Cells at a concentration of 10^6^ cfu/ml were then inoculated onto *D. pulchra* thalli in triplicate in 5 ml microwell plates. The plates were incubated at 25°C with shaking at 25 rpm for 5 d and analysed using an Olympus BX5OF-3 light microscope. At least five fields of view at 10- and 45-fold magnification were examined for the presence of biofilms, invasion of cells and bleaching of the alga. Invasion was defined as the presence of intra-cellular bacteria within algal cells and was assessed by observing bacteria moving around within the confines of the algal cell. Bleaching was defined as a loss of photosynthetic pigments (red) in algal cells, when colonized by bacterial biofilms.

### Indoleacetic acid (IAA) detection

For detection of IAA synthesis in strain R11, a 50 ml test tube containing 5 ml of yeast, tryptone and sea salt (½ YTSS) medium (l^−1^: 2 g yeast extract, 1.25 g tryptone, 20 g sea salt) was inoculated with strain R11 and grown for 24 h at 30°C on a horizontal rotating drum fermenter. Half a millilitre of this culture was then used to inoculate 50 ml of ½ YTSS medium in a 500 ml Erlenmeyer flask, which was incubated at 160 rpm and 30°C for 10 d. After 10 d, the late stationary phase culture was extracted once with an equal volume of ethyl acetate (EtOAc) containing 0.1% formic acid, dried *in vacuo*, resuspended in methanol and analyzed by high-performance liquid chromatography-mass spectrometry (HPLC-MS). HPLC-MS analysis was performed on an Agilent 1200 Series HPLC system equipped with a diode array detector and a 6130 Series ESI mass spectrometer using an analytical Phenomenex Luna C18 column (5 µm, 4.6×100 mm) operating at 0.7 ml/min with a gradient of 10% MeCN in H_2_O (plus 0.1% formic acid) to 100% MeCN over 25 min. Authentic IAA (Sigma-Aldrich, St. Louis, MO, USA) was analyzed under identical conditions.

## Results and Discussion

### Metabolic adaptation to planktonic and surface-associated marine environments

**Figure 1:**
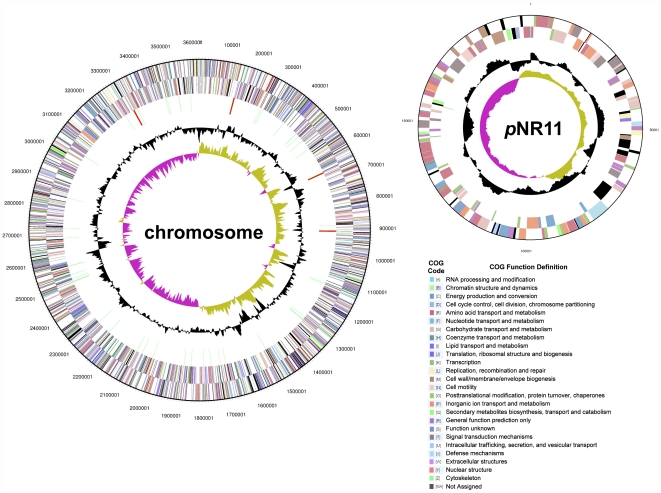
*Nautella* sp. R11 chromosome and plasmid pNR11. From outside to the center: Genes on forward strand, reverse strand (color by COG categories as indicated), rRNA genes (tRNAs green, rRNAs red, other RNAs black), GC content and GC skew.

The complete genome of *Nautella* sp. R11 is composed of a 3,622,063 bp chromosome and a 197,683 bp plasmid, termed pNR11 (Figure 1). The R11 genome encodes a total of 3,499 predicted proteins, of which 3395 genes are chromosomally encoded and the remaining 174 genes are encoded by the plasmid. A putative function could be assigned to 83.58% of these genes and 14.46% encode hypothetical proteins (Table 2).

**Table 2:** General features of the genomes of strains R11 and LSS9.

Feature	*Nautella* sp. R11	*Phaeobacter sp. LSS9 (draft)*
DNA G+C Percentage	60.01%	60.31%
16S rRNA genes	4	1 (repeats not resolved)
tRNA genes	58	49
Total number of protein coding genes	3499	3116
Genes with function prediction (% of Total Proteins)	83.58%	71.80%
Genes without function prediction (% of Total Proteins)	14.46%	26.50%

Strain R11 has the complete set of biosynthetic pathways for glycolysis, the pentose phosphate pathway and the tricarboxylic acid cycle that are characteristic of heterotrophic bacteria, as well as the pathways required for biosynthesis of nucleotides and all 20 amino acids. The R11 genome also shows genomic evidence for lithoheterothrophic growth, as it possesses the *sox* gene cluster (*soxRSVWXYZABCD*) for the oxidation of sulphur compounds. The oxidation of reduced sulphur compounds, which are often found in marine micro-niches, would provide an extra energy source for the organism, potentially stimulating growth in a similar manner to thiosulphate enhancing the cell yield of *Ruegeria pomeroyi* by 45% [21], [35], [36]. Furthermore, R11 can potentially assimilate CO_2_ through an anaplerotic pathway involving pyruvate carboxylase (2500586384) and pyruvate-phosphate dikinase (2500586764), similar to that proposed for *Roseobacter denitrificans* [37]. Together, lithoheterotrophic growth and CO_2_ fixation may allow strain R11 to utilize several readily available inorganic compounds as energy sources, and safeguard anabolic processes in situations of limited organic carbon availability. This might be particularly relevant for survival during a planktonic stage in oligotrophic waters.

In contrast, life in the phycosphere of *D. pulchra* represents a very different nutritional environment and strain R11 also shows metabolic adaptations for the uptake and utilization of readily available algal metabolites. The R11 genome possesses transporters and a demethylase (DmdA 2500585954) for the uptake and degradation of the abundant algal osmolyte dimethyl sulfoniopropionate (DMSP) [38], [39], [40]. Several transporters for typical components of algal cytosols such as glyoxylate, taurine, glycine betaine, polyamines, organic acids, acetate, branched-chain amino acids and arginine (Table S1) were found, supporting the proposition that strain R11 can effectively utilize exudates from algal tissue.

Iron is a limiting nutrient in the marine environment, but surprisingly, no genes for the synthesis of siderophores could be detected in the R11 genome. Instead strain R11 possesses a homolog of the gene *viuB* (2500584731) for the utilization of the siderophore vibriobactin and this could facilitate scavenging from co-colonizing *Vibrio* species, which are ubiquitous in the marine environment [41]. In addition, two genes for heme-binding proteins (Table S2) were found on the plasmid of R11 suggesting that the strain could meet its iron requirements from the heme-rich photosystems of its algal host [42], [43], [44]. Strain R11 also has a variety of acquisition mechanisms for phosphorous, another limiting growth factor. The genome encodes for a high-affinity phosphate transport system as well as a phosphonate transport system for organic phosphates. The presence of the polyphosphate kinase gene also suggests that an intracellular supply of polyphosphate is maintained.

Together these metabolic and physiological properties are consistent with the metabolic versatility that is observed in other members of the Roseobacter clade, traits that enable Roseobacters to become very abundant and highly competitive in bacterial communities associated with marine algae [34], [45]. We suggest that similar traits in strain R11 confer competitive advantages to life in planktonic and algal surface-associated stages.

### Attachment, colonization and persistence on algal surfaces

Motility and chemotaxis are important for virulence of several pathogenic bacteria [46], [47], [48], [49], [50]. Motility was confirmed experimentally for strain R11 [51] and all the structural genes for flagella biosynthesis were found in the genome. In addition, R11 possesses 14 chemotaxis receptors and several signal transducers, indicating that the strain can respond chemotactically to a large array of attractants or repellents. Strain R11 may also move phototactically to light by means of a two-component red-light sensing phytochrome system and a one-component BLUF domain signal transduction system [52], [53]. Together these features may enable planktonic R11 cells to orient themselves in marine waters and seek out nutrient-rich algal surfaces.

An important feature in bacterial pathogenesis is the ability of bacteria to adhere to surfaces and colonize them in the form of biofilms [54], [55]. The adhesion of bacteria to host cells by means of cell surface adhesins is often the first step in the initiation of disease [56] and fimbrial adhesins are crucial virulence factors in both plant and human pathogens [57]. Several genes related to the assembly of Type IV pili or fimbriae were detected in the R11 genome (Table S3). In addition, the enzyme cellulose synthase (2500584709) in strain R11 may synthesize cellulose fibrils, which could anchor the cells to the surface of *D. pulchra*. This mechanism has been described for *Rhizobium leguminosarum* and *Agrobacterium tumefaciens*, where cellulose fibrils are required for the initial attachment to host cells [58], [59].

After initial attachment to the host, the next step in pathogenesis is host surface colonization. Bacteria affiliated with the Roseobacter clade are efficient colonizers of marine surfaces [60] and *P. gallaeciensis*, which is phylogenetically closely related to strain R11, is capable of invading and displacing the pre-established biofilm of bacteria on the marine green alga *Ulva australis* [61]. Strain R11 forms biofilms both *in vitro* [51] and on the surface of *D. pulchra* [7] and its genome encodes proteins related to the regulation and synthesis of exopolysaccharides (EPS) (Table S4). EPS is also involved in the suppression of plant defence mechanisms and is required for infection in the rhizobia-legume symbiosis [62], [63].

The surface microbiota of marine algae and other living surfaces can prevent colonization by other bacteria through mechanisms such as the production of inhibitory molecules, competition for space and rapid utilization of available nutrients [64], [65], [66]. Numerous (31 genes) permeases of the drug/metabolite transporter (DMT) superfamily, multidrug efflux pumps and drug resistance proteins are encoded in the R11 genome (Table S5) and could protect the strain from the antibiotics and toxins secreted by competing microbiota. In addition, ABC-type antimicrobial peptide transport systems could be involved in the export of yet-to-be-identified peptides for inhibiting the growth of surrounding bacteria.

In photosynthetic organisms, reactive oxygen species are continuously being produced either as a result of photosynthesis or as a defence mechanism against microbial invasion [67]. Oxidative stress caused by reactive oxygen species such as hydrogen peroxide, organic peroxides and superoxide can directly kill a colonizing microorganism as well as trigger a systemic plant defence response [68], [69]. In order to successfully persist on plant tissues, bacteria must possess the ability to protect themselves from oxidative stress [70]. Thus, enzymes that neutralize reactive oxygen species function as virulence factors in several well-characterized phytopathogens [71], [72]. The genome of strain R11 encodes for the enzymes superoxide dismutase, catalase/peroxidase, glutathione peroxidase, a hydroperoxide resistance regulatory protein and four peroxidase-related proteins (Table S6), which together may provide cells with resistance to the highly oxidizing micro-environment of algal tissue.

### Potential virulence mechanisms involved in bleaching and invasion

A range of potential virulence factors was identified in the R11 genome, several of which were plasmid encoded (Table S2). None of the putative virulence genes were clustered in apparent genomic island nor were they associated with the only three putative transposase genes (described by PFAM 01609) present in the genome. Nevertheless, these genome-encoded virulence factors could, in concert or independently, lead to the phenotypic characteristics of bleaching and invasion of *D. pulchra* by mechanisms that include inhibition of photosynthesis, cytolytic toxins, intracellular invasion as well as the suppression of the alga's defence system.

#### Inhibition of photosynthesis

Urea is formed in the environment by bacterial degradation of nucleic and amino acids and is ubiquitous in marine ecosystems [73]. Strain R11 possesses all subunits for a secreted urease that hydrolyzes urea to CO_2_ and ammonia. Ammonia in turn is an important source of nitrogen, but is also a potent inhibitor of photosynthesis. By passing through thylakoid membranes, ammonia short circuits the pH gradient across the membrane thus uncoupling photosynthesis in chloroplasts. This inhibition of photosynthesis by ammonia has been proposed to be a mechanism of coral bleaching by the pathogen *Vibrio shilonii* [74], whose virulence is targeted at the coral's intracellular zooxanthellae. The phenotypic effects of bleaching in *D. pulchra* mimic the loss of photosynthetic function, thus making ammonium-based inhibition of photosynthesis a possible mechanism for bleaching by strain R11. Urease is absent from the genome of the non-bleaching Roseobacter, *P. gallaeciensis* BS107, which is the same species as the urease-producing strain LSS9 that causes bleaching in *D. pulchra* (see below and Table 3).

**Table 3:** Roseobacter genomes with AHL-driven quorum-sensing networks and other genes encoding putative virulence mechanisms (^B^ indicates strains with ability to cause bleach).

Organism	No of LuxI Homologs COG3916	No of LuxR Homologs pfam03472	Cellulose Synthase	Flp Pilus	Auxin Indoleacetamide Hydrolase EC:3.5.1.4	Auxin Nitrile Hydratase EC:4.2.1.84	Urease EC: 3.5.1.5	RTX Toxins	Succinoglycan Biosynthesis	Nodulation Protein L
*Nautella* sp. R11 ^B^	2	3	1	+	**+**	**+**	**+**	**+**	**+**	**+**
*Phaeobacter* sp. LSS9^B^	2	3	0	+	**+**	**+**	**+**	**+**	**+**	**+**
*Dinoroseobacter shibae* DFL 12	3	6	0	0	**−**	**+**	**+**	**+**	**−**	**−**
*Maricaulis maris* MCS10	0	0	0	+	**−**	**−**	**−**	**−**	**−**	**−**
*Oceanicaulis alexandrii* HTCC2633	0	1	0	+	**−**	**−**	**−**	**−**	**−**	**−**
*Oceanicola batsensis* HTCC2597	0	1	0	0	**+**	**+**	**+**	**−**	**−**	**−**
*Oceanicola granulosus* HTCC2516	1	3	0	0	**−**	**−**	**+**	**−**	**−**	**−**
*Phaeobacter gallaeciensis* 2.10	2	4	0	0	**+**	**+**	**+**	**+**	**+**	**+**
*Phaeobacter gallaeciensis* BS107	2	3	0	0	**+**	**+**	**−**	**+**	**+**	**+**
*Rhodobacter sphaeroides* 2.4.1	1	7	3	+	**+**	**−**	**+**	**+**	**+**	**−**
*Rhodobacter sphaeroides* ATCC 17025	1	6	4	+	**+**	**−**	**+**	**−**	**−**	**−**
*Rhodobacter sphaeroides* ATCC 17029	1	6	0	+	**+**	**−**	**+**	**−**	**+**	**−**
*Rhodobacterales bacterium* HTCC2654	2	4	0	+	**−**	**−**	**+**	**+**	**−**	**−**
*Roseovarius nubinhibens* ISM	1	2	1	0	**+**	**−**	**−**	**−**	**−**	**−**
*Roseovarius* sp. HTCC2601	0	3	0	+	**+**	**−**	**+**	**+**	**+**	**+**
*Ruegeria pomeroyi* DSS-3	2	4	0	0	**+**	**+**	**+**	**−**	**−**	**−**
*Ruegeria* sp. TM1040	0	4	0	+	**+**	**+**	**+**	**+**	**−**	**−**
*Sulfitobacter* sp. EE-36	1	2	0	0	**+**	**+**	**+**	**−**	**−**	**−**

#### Cytolytic toxins

In addition to hemolysin and several proteins with hemolysin-type calcium-binding regions, several genes encoding putative RTX toxins were detected in the R11 genome (Table S7). These include leukotoxins and FrpC proteins, believed to be virulence factors in *Neisseria meningitides* [75]. RTX toxins are a family of cytolytic pore-forming protein exotoxins that function as important virulence factors in a wide range of pathogenic Gram-negative bacteria [76], [77]. Host cell lysis may be one mechanism used by strain R11 to gain access to nutrients available within the algal cell. These cytolytic exotoxins secreted by strain R11 may function as additional virulence factors that lead to loss of cell structure and subsequent bleaching.

#### Intracellular invasion

**Figure 2:**
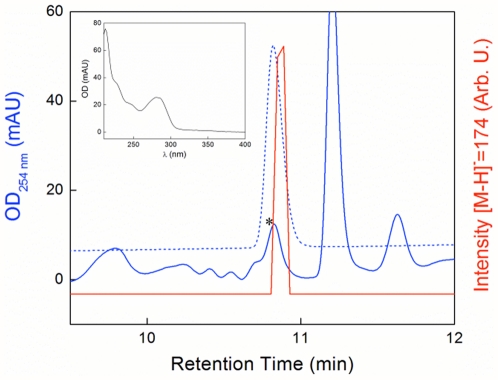
HPLC-MS analysis of *Ruegeria* strain R11 cultures extracted with acidified EtOAc. The extract was separated on a C18 column, and the elution profile, monitored at 254 nm, is shown (blue trace). At 10.8 min, a peak (black star) eluted with an absorption spectrum (inset) similar to that of IAA. The elution profile of authentic IAA (dotted blue trace), and an ion-extracted trace with the negative ion mass of 174, indicative of IAA, is also shown (red trace).

Strain R11 does not use the infection strategy of nectrotropic pathogens that penetrate plant tissue and gain access to nutrients by means of cell-wall degradation. In agreement with the microscopic observation of the disease progression [7], no cell-wall degrading enzymes could be detected in the R11 genome. Strain R11 however possesses a biosynthetic pathway from indole-3-acetonitrile to the phytohormone indole-3-acetic acid (IAA). This pathway is catalyzed by the enzymes indoleacetamide hydrolase, nitrile hydratase alpha and beta subunits (2500586135, 2500585078 and 2500585079) [78], [79]. We experimentally detected IAA by chemical analysis (Figure 2) directly supporting this function and showed IAA production by a member of the Roseobacter lineage. Phytohormones or auxins such as IAA play a critical role in plant growth and development [80]. Furthermore, IAA is secreted by several commensal and phytopathogenic bacteria [81] and functions as a virulence determinant in the phytopathogens *Pseudomonas savastanoi*, *Agrobacterium tumefaciens* and *Agrobacterium rhizogenes* [82]. It has been proposed that secretion of IAA by phytopathogenic bacteria creates an auxin imbalance to induce cell hypertrophy and loosens cell walls, which would enable bacteria to access plant tissues and intracellular nutrients [82], [83], [84]. The presence of IAA in strain R11 might therefore be a factor that contributes to its invasion of algal tissue.

Four genes (2500587620, 2500587622, 2500587626 and 2500587492) provide the full complement of genes required for the biosynthesis, transport and export of succinoglycan. Succinoglycan is an acidic exopolysaccharide polymer, which is crucial for the successful invasion of nodules and establishment of symbiosis by *Rhizobium meliloti*. Succinoglycans are thought to function as specific signal molecules that ensure the successful entry of bacteria into developing nodules. Mutants that do not produce succinoglycans are symbiotically defective and cannot initiate the formation of infection threads [85]. Succinoglycan synthesis may therefore be an additional factor that allows strain R11 to penetrate algal cells.

#### Suppression of host defence responses

Plants possess innate and adaptive resistance mechanisms to combat pathogen invasion. Upon breaching morphological barriers and coming into contact with host recognition systems, invading bacteria activate the plant's hypersensitive response by inducing pathogenesis-related (PR) defence proteins [86]. The plant's hypersensitive response is characterized by an oxidative burst and programmed cell death of host cells in the local region surrounding an infection. The PR response arrests the spread of the invading pathogen [87]. Nevertheless, pathogens have evolved several strategies that suppress the induction of plant defence responses [88]. In addition to its oxidative stress resistance genes (see above and Table S6), the R11 genome also encodes a homolog of the nodulation protein L (2500585294). In *Rhizobium* sp. NGR234, this protein negatively modulates signal transduction pathways that activate PR proteins thus suppressing the innate plant hypersensitive defence response [89], [90]. The nodulation protein L has also been shown to increase susceptibility to pathogen attack when expressed in tobacco plants [90]. Thus, through the inhibition of host PR proteins and EPS (see above), strain R11 could inhibit microbial-induced host defence reactions to facilitate its invasion of *D. pulchra* tissue.

### Regulation of virulence factor expression

A colonizing or invading bacterium must tightly control the expression of virulence factors. Two-component signal transduction systems (TCST) are one of the signalling mechanisms that enable pathogenic bacteria to adapt to different niches by sensing changes in the environment [91], [92]. Strain R11 encodes for ten TCST systems, which play a crucial role in virulence in several well-studied pathogenic bacteria (Table 4).

**Table 4:** Two-component signal transduction (TCST) systems in the R11 genome.

TCST system	Function	Organism of description	R11 protein (Gene ID)	Similarity
Gac system	Virulence master regulator	*Pseudomonas syringae*	GacS 2500587471GacS 2500584234	312/604 (51%) 187/313 (59%)
PhyR	Essential for plant colonization, regulation of a number of stress proteins	*Methylobacterium extorquens*	PhyR 2500586700	173/254 (68%)
RpfC/RpfG	Connects large-scale virulence regulation with cell-to-cell communication	*Xanthomonas campestris, Xylella fastidiosa*	RpfC 2500585385RpfG 2500585721	270/552 (48%) 116/213 (54%)
BvgS/BvgA	Expression of toxins and other virulence factors	*Bordetella pertussis*	BvgS 2500585149	155/302 (51%)
ExoS-ChvIChvG –ChvI	Regulating the production of succinoglycan, Tumour-forming ability	*Sinorhizobium meliloti, Agrobacterium tumefaciens*	ChvG 2500584411ChvI 2500584410	320/566 (56%) 188/238 (78%)
PfeR/PfeS	Expression of the ferric enterobactin receptor	*Pseudomonas aeruginosa*	PfeS 2500586717	63/132 (47%)
QseB/QseC	Interkingdom cross-signaling AI-3 quorum sensing system	*Haemophilus influenzae, Escherichia coli* (EHEC)	qQseC 2500586588	156/316 (49%)
OmpR-EnvZ	Virulence of *Shigella flexneri*, Vi polysaccharide synthesis in *Salmonella typhi*	*Shigella flexneri, Salmonella enterica*	EnvZ 2500586010	149/265 (56%)
PecS/PecM	Virulence-factor synthesis in *Erwinia chrysanthemi*	*Erwinia chrysanthemi*	PecS 2500584665PecM 2500584664	97/149 (65%) 163/249 (65%)
VirA/VirG	Tumorgenesis	*Agrobacterium tumefaciens*	VirA 2500585820 VirG 2500587202	261/431 (60%) 129/229 (56%)

In addition, two LuxI-type AHL synthases were detected in the R11 genome, along with three LuxR-type transcriptional regulators. This is consistent with the R11's production of two AHLs, N-octanoyl-homoserine lactone (OHL) and N-hexanoyl-homoserine lactone (HHL) [7]. Virulence mechanisms such as secondary metabolite production, motility, secretion, and biofilm formation are regulated by AHL-based quorum sensing (QS) in several pathogenic bacteria [93], [94], [95], [96].

Pathogenic bacteria often use temperature as a cue to induce virulence gene expression. Temperature is a key environmental parameter implicated in coral bleaching. This hypothesis is supported by the observation that the coral pathogens *V. shilonii* and *V. coralliilyticus* synthesize their virulence factors only in response to elevated seawater temperatures [97]. The histone-like nucleoid structuring (H-NS) protein is a key temperature-dependent regulator in *Escherichia coli* K-12 and *Salmonella enterica* serovar *typhimurium* [98], [99] and is also crucial in the virulence gene expression and pathogenicity of the plant pathogen *Erwinia chrysanthemi* [100]. A H-NS protein (2500587029) is encoded in the R11 genome and may play a role in the thermoregulation of virulence [7]. In addition, we investigated the presence of RNA thermometers, which can sense temperature changes and regulate translation via conformational changes [101]. In alpha- and gamma-proteobacteria the expression of small heat shock genes is commonly regulated by ROSE-like RNA-thermometers [102]. Accordingly, the two small heat-shock genes *ibpA* and *hspD* appear to be temperature-regulated in strain R11 as typical ROSE-like RNA thermometers motifs were recognizable in their upstream regions. No other RNA thermometers were found, suggesting that RNA thermometers are not involved in virulence gene expression.

### Comparative genomics reveals potential key virulence determinants

**Figure 3:**
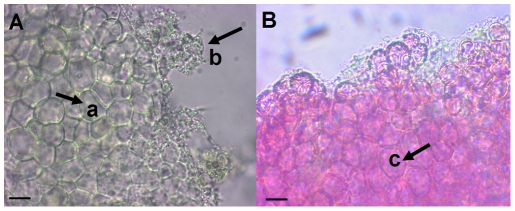
Bleaching of *Delisea pulchra* by strain LSS9. Algae were grown in artificial seawater without bromine and incubated at 25°C with 10^6^ cells/ml of strain LSS9 (**A**) and without bacteria (**B**). Bleached (pigment-free) algal cells (arrow a) and biofilms (arrow b) formed by strain LSS9 and are indicated by black arrows in (**A**). Healthy, red-pigmented algal cells (arrow c) are visible in the control algae (**B**). Scale bar  =  10 µm.

To identify potential key virulence determinants we screened 18 Roseobacter-affiliated bacteria (Table 1 and see Figure S1 for their phylogenetic relationship) for their ability to cause bleaching and/or invasion and subsequently compared their genomes for common genes. In addition to *Nautella* sp. Strain R11, only *Phaeobacter* sp. LSS9 induced the symptoms of bleaching disease in defense-deficient algae with pigment-free cells readily visible (compare Figure 3A with pigmented control tissue in panel B). However, unlike strain R11 (see Video S1), strain LSS9 did not invade *D. pulchra* cells (Figure 3). All other strains tested, while being capable of colonizing the alga under our experimental conditions, did not induce recognizable changes in *D. pulchra*. Like strain R11, strain LSS9 was also isolated from the epiphytic bacterial community on the surface of healthy *D. pulchra* [30]. We generated a draft genome sequence for strain LSS9 and used comparative genomics to identify 26 proteins that were common to the LSS9 and R11 genomes, but absent in the remaining 16 non-bleaching/non-invasive strains. Twenty of those proteins are hypothetical proteins and the six proteins with functional assignment are listed in Table 5. Of these, one protein (2500584961) was found to be a transcriptional regulator that contains autoinducer-binding and transcriptional-activator domains characteristic of LuxR-type response regulators. The LuxR-type transcriptional activators from strain R11 and strain LSS9 share 40% identity, with the next closest BLAST hit being the LuxR-type regulator from *Ruegeria pomeroyi DSS-3* with only 26% identity. Gene neighbourhood analysis demonstrated that this transcriptional regulator is an “orphan” or “solo” LuxR regulator [103], [104], since gene encoding a homolog to a LuxI type AHL synthase could be not detected in close proximity. Solo LuxR regulators have been proposed to play a key role in niche adaptation to constantly changing environments by modulating of newer, “beneficial” regulons [104]. Additionally, orphan LuxR regulators have also been proposed to eavesdrop on the signals of other bacteria [103], [105] and have been shown to respond to plant signals [106] suggesting that they have role in intra- and inter-domain communication.

**Table 5:** Unique proteins with functional annotation encoded by the genomes of strains R11 and LSS9.

Accession #	Annotation
2500584742	27 kDa antigen Cfp30B
2500584764	Uncharacterized protein Rv1520/MT1570
2500584961	Transcriptional activator protein (LuxR-type)
2500585390	Sulfotransferase family cytosolic 1B member
2500585419	Portal lambda: phage portal protein, lambda family
2500585514	Hydroxypyruvate isomerase

### Evolutionary and ecological perspectives of virulence

The occurrence of a specific LuxR-type protein in the genomes of the disease-causing pathogens strain R11 and strain LSS9, but not the remaining 18 non-disease strains examined, provides a possible explanation for the link between QS and bleaching. Many of the proposed virulence genes described here are not unique to strains R11 or LSS9 and their distribution (Table 3) appears to be typical of the ‘mix-and-match’ genome arrangement characteristic of the Roseobacter clade [34]. Despite this, the co-ordinated expression of virulence genes by the unique LuxR-type transcriptional regulator in the form of a virulence regulon may be the key determinant of pathogenicity. Global regulatory mechanisms such as QS have been implicated in the co-ordinate expression of a variety of virulence genes scattered across the bacterial chromosome and the disruption of QS circuits has been demonstrated in mutants in which virulence is down-regulated or attenuated [107], [108], [109], [110]. QS and TCST systems have also been shown to play a crucial role in the adaptation of phytopathogens to different ecological niches [111] and are thus likely to play a role in environmental adaptation by strains R11, LSS9 and other Roseobacters.

While AHL-driven quorum sensing is common to the genomes of Roseobacter strains (Table 3), it is likely to regulate different sets of genes. Recent research has shown that the transcriptional regulator PhoP, which governs virulence and magnesium homeostasis in several bacterial species, directs the expression of largely different gene sets in ten species of the *Enterobacteriaceae* [112]. Similarly, differences in virulence and host range displayed by various *S. enterica* subspecies are thought to be dictated by the specific repertoire of virulence genes acquired, along with the regulatory systems that control them in the different strains [113]. Thus global transcriptional regulation seems to reflect both regulation of species-specific targets and transcriptional rewiring of shared genes [114] and suggests that Roseobacter-affiliated strains, such as R11 and LSS9, may have evolved a virulence regulon and specific regulators that enable them to cause disease in a marine red alga.

The unique QS-dependent transcriptional regulator provides a further link between the furanone-based chemical defence of *D. pulchra* and disease. Any QS-regulated virulence regulon would be repressed by the furanones secreted by healthy *D. pulchra*, but when its chemical defence is lowered, the QS-regulated virulence regulon would be able to activate virulence factors and initiate disease. This model for virulence suppression by chemical antagonists is also supported by the observation that the QS-regulated virulence of *Pseudomonas aeruginosa* can be attenuated *in vitro* by means of synthetic furanones that are structurally similar to natural furanones isolated from *D. pulchra* [115].

It is important to note that the furanone content of *D. pulchra* is lowered by summer conditions [5]. Climate change and resulting elevated sea surface temperatures are proposed to compromise the physiological fitness of host organisms leading to ideal conditions for disease-causing bacteria to proliferate [1], [116]. Recent studies on diseased corals and sponges have also arrived at the model that latent and usually non-pathogenic commensals can turn into opportunistic pathogens under temperature stress [18], [117]. Both strains R11 and LSS9 were originally isolated from healthy *D. pulchra* and could represent such opportunistic pathogens that exploit reduced host defence to mount a QS-regulated attack.

### Conclusion

The genome of *Nautella* sp. strain R11 serves as a guide to link genetic traits to its ecology, bacteria-algae interactions, virulence and adaptation to changing environmental conditions. Strain R11 has encoded in its genome metabolic versatility consistent with an ‘opportunitroph’ strategy that has been described for other Roseobacter strains [36]. In oligotrophic environments, such as the open ocean, strain R11 could persist through its lithoheterotrophic metabolism, while chemotaxis, phototaxis and motility could facilitate its approach to nutrient rich zones such as algal surfaces. The abundance of uptake and utilization systems of algal metabolites and oxidative stress enzymes, indicate that strain R11 is well adapted to a life on algal surface communities. A breakdown of host defence mechanisms (including furanones) in a changing environment along with activation of QS-regulated virulence genes could enable strain R11 to make the transition from a commensal/symbiotic bacterium to an invading pathogen that grows and multiplies intracellularly within algal tissue, ultimately leading to bleaching and disease. Here a unique LuxR regulator identified in strains R11 and LSS9 might play a key role in the coordinated expression of virulence factors, thereby explaining how the QS inhibition by host-derived furanones can inhibit their pathogenic lifestyle.

The phylogenetic group of α-proteobacteria contains bacterial species with a wide variety of lifestyles [118], including obligate intracellular (*Rickettsia*), facultative intracellular (*Bartonella*, *Brucella*), and extracellular (*Agrobacterium*) pathogens, as well as symbionts of both animals and plants (*Wolbachia*, *Sinorhizobium*), while several Roseobacter strains have been implicated in phenomena as diverse as coral disease [25], [26], gall disease in rhodophytes [27] and algicidal activity on red tide causing dinoflagellates [22]. Here we provide for a member of the Roseobacter group the first evidence for the genetic potential for intracellular invasion of eukaryotic cells and for the biosynthesis of the plant auxin IAA, a known virulence determinant in other well characterized phytopathogens.

Virulence genes are surprisingly widespread in the genomes of marine bacteria [119] and the Roseobacter clade appears to be no exception (see Table 3). While virulence genes may have alternative functions in different strains and search thresholds may bias their identification, their conservation in a wide range of bacteria does imply a selective pressure to maintain them in the genome [120]. This pressure could reflect the need to maintain a symbiotic relationship or the benefits generated from invading a eukaryotic host. In this regard it is also possible that the virulence factors identified in the genomes of strains R11 and LSS9 have roles beyond those postulated for inducing disease in *D. pulchra*, including interactions and disease in other marine higher organisms, such as invertebrates or mammals.

## Supporting Information

Figure S1 **Phylogenetic trees based on the 16S rRNA gene for Roseobacter-affiliated strains screened for the ability to cause bleaching and used for comparative genomics.** Full length 16S rRNA gene were aligned with ClustalW (Larkin MA, Blackshields G, Brown NP, Chenna R, McGettigan PA, McWilliam H, Valentin F, Wallace IM, Wilm A, Lopez R, Thompson JD, Gibson TJ, Higgins DG (2007). “ClustalW and ClustalX version 2”. *Bioinformatics* **23** (21): 2947–294) using default parameter and a maximum likelihood tree was calculated with 1000 bootstraps and default parameters with PhyML (Guindon S, Gascuel O. A simple, fast, and accurate algorithm to estimate large phylogenies by maximum likelihood. Systematic Biology. 2003 52(5):696–704.). The numbers on nodes give bootstrap values and the scale bar indicates phylogenetic distance.
(DOC)

Table S1 **Proteins for uptake and utilization of components of algal cytosols.**
(DOC)

Table S2 **Plasmid encoded proteins possibly involved in virulence.**
(DOC)

Table S3 **Proteins related to the assembly of Type IV pili or fimbriae.**
(DOC)

Table S4 **Proteins related to exopolysaccharide (EPS) synthesis.**
(DOC)

Table S5 **Transporters and resistance proteins involved in antimicrobial defence in strain R11.**
(DOC)

Table S6 **Proteins for resistance to oxidative stress.**
(DOC)

Table S7 **Cytolytic toxins.**
(DOC)

Video S1 **Bleaching and invasion of** ***Nautella sp.*** **R11 into the tissue of** ***D. pulchra*** **.** Footage was taken at 1000× magnification. Note the motile cells inside the algal cells and the transparent colour of algae due to bleaching.
(WMV)
