# Supplementary material for: Genomes and Virulence Factors of Novel Bacterial Pathogens Causing Bleaching Disease in the Marine Red Alga Delisea pulchra
Source: PLoS One. 2011 Dec 5;6(12):e27387. doi: 10.1371/journal.pone.0027387 (PMC3230580; doi:10.1371/journal.pone.0027387)
Supplement: Figure S1 — Phylogenetic trees based on the 16S rRNA gene for Roseobacter-affiliated strains screened for the ability to cause bleaching and used for comparative genomics. Full length 16S rRNA gene were aligned with ClustalW (Larkin MA, Blackshields G, Brown NP, Chenna R, McGettigan PA, McWilliam H, Valentin F, Wallace IM, Wilm A, Lopez R, Thompson JD, Gibson TJ, Higgins DG (2007). “ClustalW and ClustalX version 2”. Bioinformatics 23 (21): 2947–294) using default parameter and a maximum likelihood tree was calculated with 1000 bootstraps and default parameters with PhyML (Guindon S, Gascuel O. A simple, fast, and accurate algorithm to estimate large phylogenies by maximum likelihood. Systematic Biology. 2003 52(5):696–704.). The numbers on nodes give bootstrap values and the scale bar indicates phylogenetic distance. (DOC) [file pone.0027387.s001.doc]

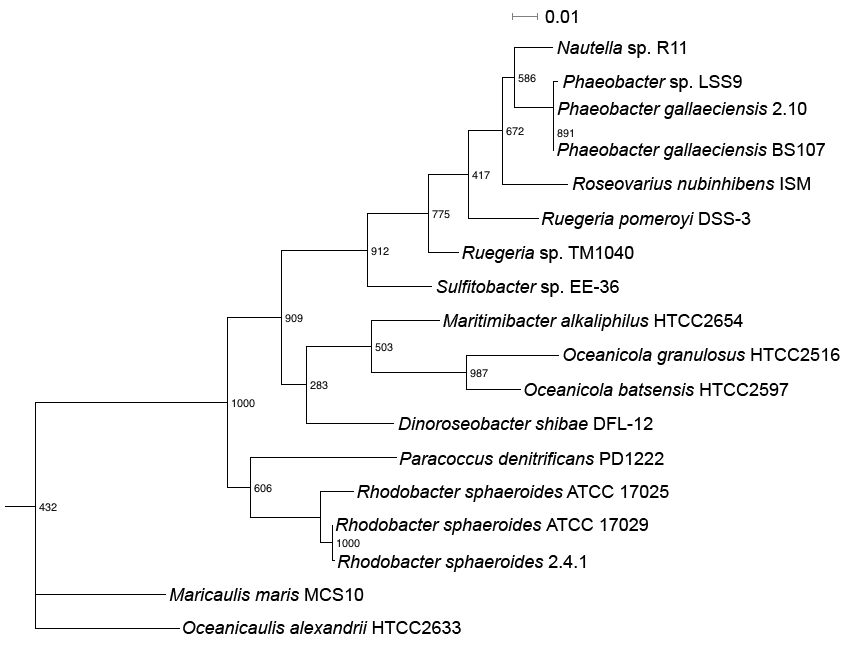


**Figure S1:** Phylogenetic trees based on the 16S rRNA gene for Roseobacter-affiliated strains screened for the ability to cause bleaching and used for comparative genomics. Full length 16S rRNA gene were aligned with ClustalW (Larkin MA, Blackshields G, Brown NP, Chenna R, McGettigan PA, McWilliam H, Valentin F, Wallace IM, Wilm A, Lopez R, Thompson JD, Gibson TJ, Higgins DG (2007). "ClustalW and ClustalX version 2". *Bioinformatics* **23** (21): 2947–294) using default parameter and a maximum likelihood tree was calculated with 1000 bootstraps and default parameters with PhyML (Guindon S, Gascuel O. A simple, fast, and accurate algorithm to estimate large phylogenies by maximum likelihood. [Systematic Biology. 2003 52(5):696-704.](http://www.ncbi.nlm.nih.gov/pubmed/14530136?ordinalpos=7&itool=EntrezSystem2.PEntrez.Pubmed.Pubmed_ResultsPanel.Pubmed_DefaultReportPanel.Pubmed_RVDocSum)). The numbers on nodes give bootstrap values and the scale bar indicates phylogenetic distance.
