# Supplementary material for: Genomes and Virulence Factors of Novel Bacterial Pathogens Causing Bleaching Disease in the Marine Red Alga Delisea pulchra
Source: PLoS One. 2011 Dec 5;6(12):e27387. doi: 10.1371/journal.pone.0027387 (PMC3230580; doi:10.1371/journal.pone.0027387)
Supplement: Table S1 — Proteins for uptake and utilization of components of algal cytosols. (DOC) [file pone.0027387.s002.doc]

**Table S1:** Proteins for uptake and utilization of components of algal cytosols

| **Accession #** | **Annotation** |
| --- | --- |
| *DMSP demethylase* | |
| 2500585954 | DMSP demethylase gene DmdA. |
| 2500584831 | Glycine betaine transporter OpuD |
| 2500587300 | Glycine betaine transporter OpuD |
| *Glyoxylate Transport* | |
| 2500584106 | Alanine-glyoxylate aminotransferase |
| 2500585069 | Serine-glyoxylate aminotransferase |
| *Taurine Transport* | |
| 2500586037 | Sulfonate/nitrate/taurine transport system sub strate |
| 2500584693 | Taurine-pyruvate aminotransferase (EC:2.6.1.77) |
| *Glycine-Betaine Transport* | |
| 2500584831 | Glycine betaine transporter OpuD |
| 2500585023 | Glycine betaine-binding protein precursor |
| 2500585024 | Glycine betaine transport system permease protein OpuAB |
| 2500585025 | Glycine betaine/L-proline transport ATP-binding protein ProV |
| 2500587575 | Betaine aldehyde dehydrogenase (EC:1.2.1.8) |
| 2500587300 | Glycine betaine transporter OpuD |
| 2500587552 | Betaine aldehyde dehydrogenase (EC:1.2.1.3) |
| 2500585207 | Betaine aldehyde dehydrogenase 1 (EC:1.2.1.8) |
| *Polyamine Transport* | |
| 2500584144 | polyamine ABC trasnporter |
| 2500584137 | Putrescine transport system permease protein PotI |
| 2500584138 | Putrescine transport system permease protein PotH |
| 2500584139 | Putrescine transport ATP-binding protein PotG (EC:3.6.3.31) |
| 2500584140 | Putrescine-binding periplasmic protein precursor |
| 2500584143 | Spermidine/putrescine import ATP-binding protein PotA (EC:3.6.3.31) |
| 2500584145 | Spermidine/putrescine transport system permease protein PotB |
| 2500586172 | Gamma-glutamylputrescine synthetase (EC:6.3.1.2) |
| 2500584294 | Putrescine-binding periplasmic protein precursor |
| 2500584295 | Spermidine/putrescine import ATP-binding protein PotA |
| 2500584296 | Spermidine/putrescine transport system permease protein PotB |
| 2500584297 | Spermidine/putrescine transport system permease protein PotC |
| 2500586170 | Gamma-glutamylputrescine synthetase (EC:6.3.1.2) |
| 2500584146 | Spermidine/putrescine transport system permease protein PotC |
| Organic Acid Transport | |
| 2500134687 | C4-dicarboxylate transport sensor protein |
| 2500135488 | TRAP dicarboxylate transporter, Dctq subunit |
| 2500135458 | TRAP dicarboxylate transporter, Dctm subunit |
| 2500135457 | TRAP dicarboxylate transporter, Dctp subunit |
| 2500135489 | TRAP dicarboxylate transporter, Dctp subunit |
| 2500136186 | TRAP C4-dicarboxylate transport system permease, Dctm subunit |
| 2500136816 | TRAP C4-dicarboxylate transport system permease, Dctm subunit |
| 2500136818 | TRAP dicarboxylate transporter, Dctp subunit |
| 2500134690 | TRAP dicarboxylate transporter, Dctp subunit |
| 2500134692 | TRAP dicarboxylate transporter, Dctm subunit |
| Acetate | |
| 2500584686 | Acetate kinase (EC:2.7.2.1) |
| Branched Chain Amino Acid Transport | |
| 2500586062 | High-affinity branched-chain amino acid transport ATP-binding protein BraF |
| 2500587088 | branched-chain amino acid transport |
| 2500585540 | High-affinity branched-chain amino acid transport ATP-binding protein BraG |
| 2500585756 | High-affinity branched-chain amino acid transport ATP-binding protein BraG |
| 2500587147 | High-affinity branched-chain amino acid transport ATP-binding protein BraG |
| 2500587193 | High-affinity branched-chain amino acid transport ATP-binding protein BraG |
| 2500584278 | High-affinity branched-chain amino acid transport ATP-binding protein LivF |
| 2500585700 | High-affinity branched-chain amino acid transport ATP-binding protein LivF |
| 2500586063 | High-affinity branched-chain amino acid transport ATP-binding protein LivF |
| 2500584279 | High-affinity branched-chain amino acid transport ATP-binding protein LivG |
| 2500585539 | High-affinity branched-chain amino acid transport ATP-binding protein LivG |
| 2500585699 | High-affinity branched-chain amino acid transport ATP-binding protein LivG |
| 2500587199 | High-affinity branched-chain amino acid transport ATP-binding protein LivG |
| 2500585537 | High-affinity branched-chain amino acid transport system permease protein BraD |
| 2500585697 | High-affinity branched-chain amino acid transport system permease protein BraD |
| 2500586060 | High-affinity branched-chain amino acid transport system permease protein BraD |
| 2500587150 | High-affinity branched-chain amino acid transport system permease protein braD |
| 2500585754 | High-affinity branched-chain amino acid transport system permease protein BraE |
| 2500587195 | High-affinity branched-chain amino acid transport system permease protein BraE |
| 2500585755 | High-affinity branched-chain amino acid transport system permease protein LivH |
| 2500587198 | High-affinity branched-chain amino acid transport system permease protein LivH |
| 2500584276 | High-affinity branched-chain amino acid transport system permease protein LivM |
| 2500585698 | High-affinity branched-chain amino acid transport system permease protein LivM |
| 2500587149 | High-affinity branched-chain amino acid transport system permease protein LivM |
| 2500587087 | Predicted branched-chain amino acid permease (azaleucine resistance) |
| 2500585757 | Probable branched-chain amino acid transport ATP- binding protein LivG |
| 2500587194 | Branched-chain amino acid ABC transporter |
| 2500587145 | Branched-chain amino acid ABC transporter |
| 2500584277 | Branched-chain amino acid ABC transporter, permease protein |
| 2500585538 | Branched-chain amino acid ABC transporter, permease protein |
| 2500587148 | High-affinity branched-chain amino acid transport ATP-binding protein BraF |
| Arginine Transport | |
| 2500586167 | Arginine-binding periplasmic protein precursor |
| 2500585852 | Twin-arginine translocation pathway signal sequence domain protein, putative |
| 2500584299 | Twin-arginine translocation pathway signal |
| 2500586166 | Arginine/ornithine transport ATP-binding protein AotP (EC:3.6.3.21) |
