# Supplementary material for: Genomes and Virulence Factors of Novel Bacterial Pathogens Causing Bleaching Disease in the Marine Red Alga Delisea pulchra
Source: PLoS One. 2011 Dec 5;6(12):e27387. doi: 10.1371/journal.pone.0027387 (PMC3230580; doi:10.1371/journal.pone.0027387)
Supplement: Table S2 — Plasmid encoded proteins possibly involved in virulence. (DOC) [file pone.0027387.s003.doc]

**Table S2:** Plasmid encoded proteins possibly involved in virulence

| **Accession #** | **Annotation** |
| --- | --- |
|  | *Toxin and related* |
| 2500587526 | Leukotoxin translocation ATP-binding protein LktB |
| 2500587600 | Leukotoxin |
|  | *Miscellaneous* |
| 2500587576 | Probable rhizopine catabolism regulatory protein MocR |
| 2500587505 | Probable multidrug resistance protein norm |
|  | Capsule and exo-polysaccharide biosynthesis |
| 2500587625 | Exopolysaccharide production protein ExoQ |
| 2500587467 | Vi polysaccharide biosynthesis protein |
| 2500587509 | Putative capsule polysaccharide export protein precursor |
|  | *Hydrolase and related* |
| 2500587629 | Beta-glucanase precursor |
| 2500587540 | GDSL-like lipase/acylhydrolase, putative |
| 2500587525 | Proteases secretion ATP-binding protein PrtD |
|  | *Adhesion* |
| 2500587620 | Succinoglycan biosynthesis protein ExoL |
| 2500587622 | Succinoglycan biosynthesis protein ExoA |
| 2500587626 | Succinoglycan biosynthesis protein ExoO |
| 2500587492 | Succinoglycan biosynthesis transport protein ExoT |
| 2500587524 | Serine-rich adhesin for platelets precursor |
|  | *Iron acquisition* |
| 2500587543 | Hemin transport system permease protein HmuU |
| 2500587545 | Hemin transport protein HmuS |
| 2500587544 | Hemin-binding periplasmic protein HmuT precursor |
| 2500587543 | Hemin transport system permease protein HmuU |
| 2500587542 | Hemin import ATP-binding protein HmuV |
| 2500587601 | Heme-binding protein A precursor |
| 2500587546 | TonB-dependent heme receptor A precursor |
