# Supplementary material for: Genomes and Virulence Factors of Novel Bacterial Pathogens Causing Bleaching Disease in the Marine Red Alga Delisea pulchra
Source: PLoS One. 2011 Dec 5;6(12):e27387. doi: 10.1371/journal.pone.0027387 (PMC3230580; doi:10.1371/journal.pone.0027387)
Supplement: Table S3 — Proteins related to the assembly of Type IV pili or fimbriae. (DOC) [file pone.0027387.s004.doc]

**Table S3:** Proteins related to the assembly of Type IV pili or fimbriae

| **Accession #** | **Annotation** |
| --- | --- |
| 2500585566 | PilZ domin |
| 2500586716 | Type 4 fimbriae expression regulatory protein PilR |
| 2500586800 | CpaB family protein; K02279 pilus assembly protein CpaB |
| 2500586809 | Flp pilus assembly protein, protease CpaA (EC:3.4.23.43) |
| 2500586804 | Flp pilus assembly protein, ATPase CpaF |
| 2500586801 | Flp pilus assembly protein, secretin CpaC |
