# Supplementary material for: Genomes and Virulence Factors of Novel Bacterial Pathogens Causing Bleaching Disease in the Marine Red Alga Delisea pulchra
Source: PLoS One. 2011 Dec 5;6(12):e27387. doi: 10.1371/journal.pone.0027387 (PMC3230580; doi:10.1371/journal.pone.0027387)
Supplement: Table S4 — Proteins related to exopolysaccharide (EPS) synthesis. (DOC) [file pone.0027387.s005.doc]

**Table S4:** Proteins related to exopolysaccharide (EPS) synthesis

| **Accession #** | **Annotation** |
| --- | --- |
| 2500585246 | Exopolysaccharide II synthesis transcriptional activator ExpG |
| 2500587625 | Exopolysaccharide production protein ExoQ |
