# Supplementary material for: Genomes and Virulence Factors of Novel Bacterial Pathogens Causing Bleaching Disease in the Marine Red Alga Delisea pulchra
Source: PLoS One. 2011 Dec 5;6(12):e27387. doi: 10.1371/journal.pone.0027387 (PMC3230580; doi:10.1371/journal.pone.0027387)
Supplement: Table S5 — Transporters and resistance proteins involved in antimicrobial defence in strain R11. (DOC) [file pone.0027387.s006.doc]

**Table S5:** Transporters and resistance proteins involved in antimicrobial defence in strain R11

| **Accession # Annotation** | |
| --- | --- |
| *DMT transporters* | |
| 2500584360 | Permeases of the drug/metabolite transporter (DMT) superfamily |
| 2500584676 | Permeases of the drug/metabolite transporter (DMT) superfamily |
| 2500586758 | Permeases of the drug/metabolite transporter (DMT) superfamily |
| 2500586627 | Predicted permease, DMT superfamily |
| 2500586757 | Permeases of the drug/metabolite transporter (DMT) superfamily |
| 2500586181 | Permeases of the drug/metabolite transporter (DMT) superfamily |
| *Drug Resistance* | |
| 2500137957 | tetracycline resistance protein |
| 2500134609 | drug resistance transporter, Bcr/CflA family |
| 2500587099 | RND_mfp: efflux transporter, RND family, MFP subunit |
| 2500134646 | glyoxalase/bleomycin resistance protein/dioxygenase |
| 2500134662 | glyoxalase/bleomycin resistance protein/dioxygenase |
| 2500134749 | glyoxalase/bleomycin resistance protein/dioxygenase |
| 2500134781 | drug resistance transporter Bcr/CflA subfamily |
| 2500135052 | tetracycline resistance protein |
| 2500585470 | Fusaric acid resistance protein FusE |
| 2500135253 | multidrug resistance efflux protein, SMR family |
| 2500135295 | drug resistance transporter, Bcr/CflA subfamily |
| 2500135561 | multiple resistance and pH regulation protein F |
| 2500136132 | glyoxalase/bleomycin resistance protein/dioxygenase |
| 2500136741 | glyoxalase/bleomycin resistance protein/dioxygenase |
| 2500137593 | glyoxalase/bleomycin resistance protein/dioxygenase |
| 2500134587 | glyoxalase/bleomycin resistance protein/dioxygenase |
| 2500587320 | Chloramphenicol 3-O phosphotransferase |
| 2500584766 | Cephalosporin hydroxylase |
| 2500586192 | Uncharacterized protein involved in methicillin resistance |
| 2500587362 | Uncharacterized bacitracin resistance protein |
| 2500584996 | Multidrug resistance protein MdtB |
| 2500585469 | Multidrug resistance protein MdtC |
| 2500587098 | Probable aminoglycoside efflux pump |
| *ABC-type antimicrobial peptide transport system* | |
| 2500585735 | ABC-type antimicrobial peptide transport system, ATPase component |
| 2500585546 | ABC-type antimicrobial peptide transport system |
