# Supplementary material for: Genomes and Virulence Factors of Novel Bacterial Pathogens Causing Bleaching Disease in the Marine Red Alga Delisea pulchra
Source: PLoS One. 2011 Dec 5;6(12):e27387. doi: 10.1371/journal.pone.0027387 (PMC3230580; doi:10.1371/journal.pone.0027387)
Supplement: Table S6 — Proteins for resistance to oxidative stress. (DOC) [file pone.0027387.s007.doc]

**Table S6:** Proteins for resistance to oxidative stress

| **Accession #** | **Annotation** |
| --- | --- |
| 2500585463 | Superoxide Dismutase |
| 2500587334 | Organic hydroperoxide resistance transcriptional regulator |
| 2500584319 | Peroxidase/catalase |
| 2500584324 | Glutathione peroxidase precursor (EC:1.11.1.9) |
| 2500585683 | Uncharacterized peroxidase-related enzyme |
| 2500584680 | Alkylhydroperoxidase AhpD family core domain |
| 2500584746 | Alkylhydroperoxidase AhpD family core domain |
| 2500584649 | Uncharacterized peroxidase-related enzyme |
