# Supplementary material for: Genomes and Virulence Factors of Novel Bacterial Pathogens Causing Bleaching Disease in the Marine Red Alga Delisea pulchra
Source: PLoS One. 2011 Dec 5;6(12):e27387. doi: 10.1371/journal.pone.0027387 (PMC3230580; doi:10.1371/journal.pone.0027387)
Supplement: Table S7 — Cytolytic toxins. (DOC) [file pone.0027387.s008.doc]

**Table S7:** Cytolytic toxins

| **Accession** | **Annotation** |
| --- | --- |
| 2500586515 | Leukotoxin |
| 2500587600 | Leukotoxin |
| 2500584160 | Leukotoxin |
| 2500585296 | Iron-regulated protein FrpC |
| 2500585553 | Iron-regulated protein FrpC |
| 2500586535 | Iron-regulated protein FrpA |
| 2500587165 | Alpha-Hemolysin translocation ATP-binding protein HlyB |
| 2500585595 | Bifunctional Hemolysin-adenylate cyclase precursor |
| 2500586304 | Hemolysin-type calcium-binding region |
| 2500584900 | Hemolysin (EC:1.13.11.27) |
| 2500587166 | Hemolysin secretion protein D, chromosomal |
| 2500134314 | Hemolysin-type calcium-binding protein |
| 2500134795 | Hemolysin-type calcium-binding region |
| 2500134858 | Hemolysin-type calcium-binding region |
| 2500135021 | Hemolysin-type calcium-binding region |
| 2500135770 | Hemolysin-type calcium-binding region |
| 2500135987 | Hemolysin-type calcium-binding region |
| 2500136007 | Hemolysin-type calcium-binding region |
| 2500136272 | Hemolysin-type calcium-binding region |
| 2500137062 | Hemolysin-type calcium-binding region |
| 2500137960 | Hemolysin-type calcium-binding region |
| 2500134698 | Hemolysin-type calcium-binding region |
